# Vitamin B12 Deficiency in Pernicious Anemia: A Hemolytic Anemia Mimic

**DOI:** 10.7759/cureus.79176

**Published:** 2025-02-17

**Authors:** Nasim Salimiaghdam, Omar Jumaah, Talar Acob, Knkush Hakobyan, Emily Chen

**Affiliations:** 1 Internal Medicine, Capital Health Regional Medical Center, Trenton, USA; 2 Internal Medicine, Capital Health Medical Center, Trenton, USA; 3 Hematology and Medical Oncology, Capital Health Regional Medical Center, Trenton, USA

**Keywords:** macrocytic anemia, pancytopenia, pernicious anemia, severe vitamin b12 deficiency, thrombocytopenia

## Abstract

Vitamin B12 deficiency can lead to a wide range of clinical symptoms and may resemble hemolytic anemia due to ineffective red blood cell production and hemolysis occurring within the bone marrow. Identifying this deficiency as a possible cause of hemolysis is essential to prevent misdiagnosis, especially when distinguishing it from thrombotic microangiopathy. We present a case involving a 60-year-old woman with a history of hypertension and type 2 diabetes who came in with symptoms of generalized weakness, dizziness, nausea, and abdominal pain. Laboratory tests showed pancytopenia, macrocytosis, and signs of hemolysis. Further investigation confirmed a severe vitamin B12 deficiency linked to pernicious anemia. After starting weekly intramuscular cyanocobalamin injections for the first month and then switching to monthly injections for four months post-discharge, her blood parameters showed significant improvement. This underlines the vital role of timely diagnosis and following established treatment protocols. This case emphasizes the importance of considering vitamin B12 deficiency as a reversible cause of hemolysis. It highlights the need to differentiate it from more serious hematologic disorders such as thrombotic microangiopathy to ensure proper management.

## Introduction

Vitamin B12 plays a vital role in the synthesis of DNA in the precursors of red blood cells and is essential for their maturation. When this vitamin is deficient, DNA synthesis is disrupted, leading to megaloblastic changes in erythroblasts. This disruption results in ineffective erythropoiesis and intramedullary hemolysis. Consequently, the inadequate production of blood cells can lead to conditions such as macrocytic anemia, pancytopenia, and various neurological issues [1,2]. In some instances, a deficiency in B12 may present symptoms similar to hemolytic anemia, including intramedullary hemolysis and elevated levels of lactate dehydrogenase (LDH), which raises concerns about serious conditions like thrombotic microangiopathy (TMA) [3,4]. TMA includes disorders such as thrombotic thrombocytopenic purpura (TTP) and hemolytic uremic syndrome (HUS), which share symptoms with B12 deficiency, such as hemolysis, low platelet counts, and the presence of schistocytes in blood smears [5,6]. However, it is important to note that anemia caused by B12 deficiency arises from ineffective erythropoiesis rather than true hemolysis, which sets it apart from TMA. In addition, other conditions like folate deficiency, myelodysplastic syndromes (MDS), hypothyroidism, and chronic alcoholism can lead to macrocytic anemia and pancytopenia, making the diagnostic process more complex [7,8]. Key distinguishing features, such as a low reticulocyte production index (RPI), hypersegmented neutrophils in cases of B12 and folate deficiencies, dysplastic changes in MDS, and macrocytosis linked to liver dysfunction in alcoholism, can aid in differentiating these causes [9]. Hypothyroidism can also contribute to macrocytic anemia by reducing erythropoiesis and causing suppression of the bone marrow [10].

In addition to blood-related complications, a deficiency in vitamin B12 can result in significant neurological issues, such as subacute combined degeneration (SCD) of the spinal cord. This condition is characterized by the demyelination of the dorsal columns and lateral corticospinal tracts, potentially leading to progressive sensory ataxia, spasticity, and proprioceptive deficits if not addressed quickly [11]. Early identification and treatment are crucial, as neurological damage may become irreversible in later stages. Misdiagnosing B12 deficiency as thrombotic microangiopathy (TMA) or other serious blood disorders can result in unnecessary procedures like plasma exchange, immunosuppressive therapy, or bone marrow biopsies, which can delay the right treatment [12].

We present a case involving a 60-year-old woman who suffered from severe macrocytic anemia and pancytopenia due to a vitamin B12 deficiency. This case highlights the urgent need for early diagnosis and intervention, which can reverse hemolytic anemia stemming from ineffective erythropoiesis, and emphasizes the importance of distinguishing it from thrombotic microangiopathy and other possible causes to ensure timely and appropriate care.

## Case presentation

A 60-year-old woman with a history of hypertension and type 2 diabetes mellitus came to the emergency department, reporting generalized weakness and dizziness that had lasted for a week. She denied experiencing chest pain, palpitations, fainting, fever, chills, rectal bleeding, or any exposure to toxins. There was no history of smoking, alcohol, or illicit drug use and no family history of blood disorders. During the examination, the patient appeared pale but was not in acute distress. Her vital signs were stable. The abdominal examination showed no abnormalities, with no organomegaly or masses detected. There were no petechiae, purpura, or signs of active bleeding. A neurological examination indicated intact cranial nerves, normal muscle strength (5/5) in all extremities, and preserved deep tendon reflexes. Sensory examination was normal, with no deficits, and coordination, gait, and Romberg testing were also unremarkable. No signs of subacute combined degeneration, such as proprioceptive loss or spasticity, were observed. The dermatologic evaluation revealed no jaundice, hyperpigmentation, or glossitis. The patient’s mental status was intact, with no signs of confusion, memory issues, or mood disturbances.

The patient presented with primary symptoms of weakness and dizziness, indicating a possible hematologic issue. The absence of chest pain, palpitations, and other specific cardiovascular symptoms made a heart-related cause less likely. In addition, the lack of fever and chills helped rule out infections, while the absence of gastrointestinal symptoms, such as rectal bleeding, suggested that an acute gastrointestinal hemorrhage was unlikely. Physical examination revealed pallor and no signs of active bleeding, raising concerns about anemia, especially considering the patient's chronic medical conditions.

Laboratory tests showed severe pancytopenia, with the following results: hemoglobin (Hb): 3.3 grams per deciliter (g/dl), white blood cell (WBC) count: 1.72 × 10^9^/L, platelet count: 28 × 10^9^/L, mean corpuscular volume (MCV): 100 femtoliters (fL), and absolute neutrophil count (ANC): 1.07 per microliter of blood (muL). There were elevated indirect bilirubin of 3.8 milligrams per deciliter (mg/dl) and high lactate dehydrogenase (LDH) of 7228 units per liter (U/L) (index 120-246), indicating hemolysis and ineffective hematopoiesis. Further workup showed an elevated reticulocyte count of 3.5% and decreased haptoglobin <10 milligrams per deciliter (mg/dL) (index: 37-355).

The anemia workup showed low vitamin B12 levels (<159 picograms per milliliter (pg/mL)) alongside normal folate (3.17 nanograms per milliliter (ng/mL)), and iron studies (iron level: 153 micrograms per deciliter (mcg/dL) and ferritin level 149.35 nanograms per milliliter (ng/mL)). Elevated antibodies to intrinsic factor (6.5 arbitrary units per milliliter (AU/mL)) confirmed a diagnosis of pernicious anemia, while a negative Coombs test ruled out autoimmune hemolysis. The peripheral blood smear indicated macrocytosis and hypersegmented neutrophils, which are classic signs of vitamin B12 deficiency. The elevated indirect bilirubin and low haptoglobin (<10 mg/dL) milligrams per deciliter (mg/dL) (index: 37-355) suggested ineffective erythropoiesis, a key feature of vitamin B12 deficiency. The increased MCV and presence of hypersegmented neutrophils further reinforced the diagnosis, supported by the patient’s clinical presentation and lab results. Although the reticulocyte count was 3.5%, the calculated reticulocyte production index (RPI) was <2, consistent with ineffective erythropoiesis rather than a truly regenerative response, further supporting vitamin B12 deficiency as the primary cause.

Management and treatment response

The patient received three units of packed red blood cells (RBCs) and started weekly intramuscular cyanocobalamin injections (1 mg for one month). At the time of discharge, her hemoglobin had risen to 9.4 g/dL, and her hematologic parameters were fully restored. She received weekly cyanocobalamin injections for one month after being admitted to the hospital and then continued with monthly injections for the next four months following her discharge. Her blood counts were monitored closely, with normalization shown in Table 1.

**Table 1:** Hematologic response to PRBC transfusion and B12 therapy PRBC: packed red blood cell, WBC: white blood cells, RBC: red blood cells, Hb: hemoglobin, MCV: mean corpuscular volume, PLT: platelets PRBC transfusion: An immediate increase in hemoglobin (6.0 g/dL) was noted on day 1 after the transfusion, which is a common response to PRBC transfusion. However, other hematologic parameters, including reticulocyte count and bilirubin levels, did not significantly improve. Vitamin B12 therapy: A steady increase in hemoglobin (reaching 9.4 g/dL by day 7) was recorded following the start of intramuscular cyanocobalamin. The reticulocyte count, MCV, bilirubin, and LDH levels gradually improved, indicating ongoing recovery attributed to vitamin B12 supplementation. Long-term monitoring: By the time of discharge (one month later), the patient's blood counts had returned to normal, reflecting both a correction in hemoglobin and other hematologic parameters, which suggests a lasting response to vitamin B12 therapy. This table clearly illustrates the immediate effects of PRBC transfusion (increase in hemoglobin) compared to the slower but sustained improvement seen with B12 supplementation (recovery of all hematologic parameters).

Time point	WBC (x10^9^/L)	RBC (x10^12^/L)	Hb (g/dL)	MCV (fL)	Platelets (x10^9^/L)
Normal range	4.0-11.0	4.7-6.1 (M) / 4.2-5.4 (F)	13.8-17.2 (M) / 12.1-15.1 (F)	80-100	150-450
1st day of admission	1.72	1.03	3.3	100	28
4th day of admission	2.37	2.50	7.8	93.6	22
Discharge	3.47	3.10	9.4	94.2	64
Four-month follow-up	4.61	4.65	12.6	86.2	165

This case highlights the significance of identifying the clinical and laboratory signs of vitamin B12 deficiency. The patient's symptoms of weakness and dizziness, linked to severe anemia, along with her lab findings-pancytopenia with macrocytosis, hypersegmented neutrophils, elevated indirect bilirubin, and low vitamin B12 levels-underscore the typical indicators of pernicious anemia. The swift response to vitamin B12 treatment serves as a crucial diagnostic signal for healthcare providers, confirming the diagnosis and illustrating the potential for quick hematologic recovery.

## Discussion

This case demonstrates significant macrocytic anemia, pancytopenia, and elevated LDH levels alongside critically low vitamin B12, indicating that vitamin B12 deficiency is likely the primary cause [13]. Several factors can lead to B12 deficiency, such as insufficient dietary intake, pernicious anemia, or absorption issues [14,15]. The absence of gastrointestinal symptoms and negative tests for hepatitis and HIV made malabsorption less probable. The presence of high levels of intrinsic factor antibodies suggests an autoimmune-related vitamin B12 deficiency, which is most consistent with pernicious anemia resulting from atrophic gastritis [16].

Further tests, including intrinsic factor antibody levels, were suggested for confirmation. Although elevated LDH and indirect bilirubin levels pointed toward hemolysis, the absence of schistocytes in the peripheral smear and her positive response to B12 injections suggested ineffective hematopoiesis instead of active hemolysis. Intramedullary hemolysis, which occurs in megaloblastic anemia due to the formation of defective red blood cells (megaloblasts), leads to increased LDH and indirect bilirubin levels. These megaloblasts are destroyed in the bone marrow before they can mature and enter the bloodstream, resulting in ineffective erythropoiesis. This situation worsens in vitamin B12 deficiency due to the impaired maturation of erythrocyte precursors. In this instance, the elevated indirect bilirubin levels and decreased haptoglobin indicated ineffective erythropoiesis rather than active hemolysis [17].

**Figure 1:**
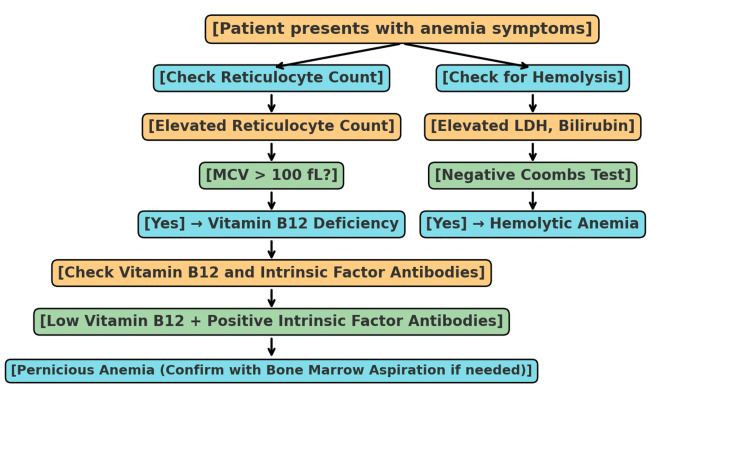
Diagnostic approach to vitamin B12 deficiency vs. hemolytic anemia This flowchart helps differentiate vitamin B12 deficiency, especially pernicious anemia, from hemolytic anemia through a combination of clinical signs, lab work, and diagnostic tests (e.g., reticulocyte count, Coombs test, LDH, bilirubin levels, and vitamin B12 testing).

The diagnostic method for differentiating vitamin B12 deficiency from hemolytic anemia involves a mix of lab results and clinical symptoms. The flowchart below outlines the essential steps in this differentiation process (Figure 1).

The peripheral blood smear showed macrocytosis (increased MCV > 100 fL) and hypersegmented neutrophils, both of which are classic indicators of vitamin B12 deficiency. These observations reinforced the diagnosis of pernicious anemia, confirmed by the presence of elevated intrinsic factor antibodies. The absence of schistocytes and a normal Coombs test ruled out autoimmune hemolysis as a potential cause. These smear results align with the disruption in normal red blood cell maturation and the resulting ineffective hematopoiesis associated with vitamin B12 deficiency [18].

There have been instances of vitamin B12 deficiency that present with pancytopenia and hemolytic features. For instance, Hughes et al. described a case of a patient suffering from severe macrocytic anemia and indirect hyperbilirubinemia who was initially misdiagnosed with hemolysis until the B12 deficiency was recognized [19]. These examples underscore the necessity of considering B12 deficiency when diagnosing hemolytic anemia.

In severe cases of B12 deficiency, macrocytic anemia typically shows hypercellular bone marrow and ineffective erythropoiesis, where immature red blood cells die off before they can enter the bloodstream [1]. While a lack of vitamin B12 can lead to serious neurological problems, this patient's quick response to treatment and absence of neurological symptoms suggested a favorable prognosis. Her hemoglobin levels improved significantly after transfusion and B12 supplementation, with blood parameters continuing to normalize during her hospital stay.

It is important to distinguish between the immediate effects of transfusion and the long-term advantages of B12 therapy. Transfusion offers a quick boost in circulating red blood cells, which temporarily enhances oxygen delivery and alleviates symptoms, but it does not address the root cause of anemia [20].

Managing vitamin B12 deficiency in cases of pernicious anemia requires lifelong B12 supplementation, as the autoimmune destruction of gastric parietal cells results in an irreversible lack of intrinsic factors. The standard treatment involves intramuscular cyanocobalamin (1 mg weekly for four weeks, then monthly for life) to prevent the recurrence of hematologic and neurological issues [21]. Alternatively, for patients who prefer oral treatment and have sufficient absorption, high-dose oral cyanocobalamin (1-2 mg daily) may be considered, although its effectiveness in pernicious anemia is still debated [22]. Due to the increased risk of gastric cancer associated with pernicious anemia, routine screenings and ongoing follow-up with a gastroenterologist are advised [23]. Research shows that these patients face a 2- to 3-fold increased risk of gastric adenocarcinoma and type I gastric carcinoid tumors linked to chronic atrophic gastritis and achlorhydria. Endoscopic monitoring may be necessary, particularly for those with additional risk factors like persistent dyspepsia, weight loss, or a family history of gastric cancer. Regular neurological and hematologic assessments are crucial, as subacute combined degeneration of the spinal cord can develop gradually even after blood parameters have normalized [24]. Patients should have routine complete blood counts (CBCs), vitamin B12 level checks, and neurological evaluations to facilitate early detection of potential complications and ensure effective management. Although B12 deficiency is not uncommon, this case highlights the need to identify its hematologic signs and differentiate ineffective erythropoiesis from actual hemolysis. Ongoing monitoring and suitable management approaches are essential to prevent long-term complications and achieve a positive outcome.

## Conclusions

This case demonstrates that a deficiency in vitamin B12 can lead to severe macrocytic anemia and pancytopenia, both of which are significant yet treatable conditions. Early detection is crucial to avoid misdiagnosis, especially when distinguishing it from thrombotic microangiopathy and other types of hemolytic anemia. A thorough evaluation is necessary to diagnose macrocytic anemia and to exclude other potential causes like myelodysplastic syndrome, folate deficiency, and liver disease. While a bone marrow biopsy was not conducted in this instance, it is generally not required for diagnosing vitamin B12 deficiency unless there is a concern for hematologic malignancies. Notably, the lack of schistocytes and a normal Coombs test support the conclusion that the anemia resulted from ineffective erythropoiesis rather than actual hemolysis. This case highlights the importance of timely treatment, such as administering vitamin B12, to avert possible neurological complications, underscoring the critical need for early diagnosis. In addition, it is vital to recognize that patients with pernicious anemia will require lifelong vitamin B12 supplementation to prevent the recurrence of hematologic and neurological problems. Thus, this case reinforces the necessity for swift identification and effective management to ensure favorable patient outcomes.
